# High-precision multiparameter estimation of mechanical force by quantum optomechanics

**DOI:** 10.1038/s41598-022-20150-6

**Published:** 2022-09-26

**Authors:** László Ruppert, Andrey Rakhubovsky, Radim Filip

**Affiliations:** Department of Optics, Palacky University, 17. listopadu 12, 77 146 Olomouc, Czech Republic

**Keywords:** Optical metrology, Quantum optics, Quantum metrology

## Abstract

A nanomechanical oscillator can be used as a sensitive probe of a small linearized mechanical force. We propose a simple quantum optomechanical scheme using a coherent light mode in the cavity and weak short-pulsed light-matter interactions. Our main result is that if we transfer some displacement to the mechanical mode in an initialization phase, then a much weaker optomechanical interaction is enough to obtain a high-precision multiparameter estimation of the unknown force. This approach includes not only estimating the displacement caused by the force but also simultaneously observing the phase shift and squeezing of the mechanical mode. We show that the proposed scheme is robust against typical experimental imperfections and demonstrate the feasibility of our scheme using orders of magnitude weaker optomechanical interactions than in previous related works. Thus, we present a simple, robust estimation scheme requiring only very weak light-matter interactions, which could open the way to new nanomechanical sensors.

## Introduction

Optical sensing of mechanical motion has fully moved to the quantum domain^1–3^. Sensing is important for applications in which a mechanical force enhances imaging accuracy in microscopy^4–6^. Microscopic force detection is advantageous in fundamental physics, chemistry, medicine and biology^7–10^, so each of these fields can benefit from developments in mechanical sensing. We will consider a small force that can both displace and squeeze mechanical motion in position and momentum variables. Mechanical displacement and squeezing not only provide information about nanomechanical processes, they have also become important resources in the field of quantum thermodynamics^11–14^. To reliably recognize their presence and potential, all aspects of a weak force *F*(*q*) linearized around any position have to be simultaneously estimated.

In quantum optomechanics^15^, the main focus has been so far on estimating the phase shift and the mechanical displacement. For a review of the field, see the work by Li et al.^16^ and references therein. Historically, there was an interest in the high-precision measurement of the displacement of a mechanical probe for the detection of gravitational waves^17,18^. The precise measurement of a mechanical displacement has a high scientific merit in itself^19–21^ and is of paramount importance in force microscopy^4–6,22^, magnetometry^23–25^ and the probing of fundamental quantum effects at macroscopic scales^3,26^, to mention a few. Force sensing via ultraprecise displacement detection is an important direction of research in optomechanics^6,27–31^. It is, however, incomplete. From a theoretical point of view, a linear effect of an unknown weak deterministic force is generally equivalent to a Gaussian unitary process simultaneously displacing and squeezing the oscillator. There are five parameters of a general Gaussian unitary transformation^32^, therefore the problem of force detection automatically translates to a multiparameter estimation problem. Ignoring some of these parameters is conceptually inaccurate, it results in misleading conclusions and can underestimate resources. It is therefore necessary to estimate each parameter of the process by applying fast short-time optomechanical interactions and measuring the state of the light mode.

The estimation of special cases of Gaussian processes is widely studied in the literature. The estimation of the displacement is usually performed by simple homodyne measurements^33^. The most popular subject is the estimation of the phase shift, which is solved using interferometry^34^. The task of estimating the squeezing is of similar difficulty, but it has received much less attention in the past^35–38^. This problem is usually solved for general settings, but there are also some results for specific systems^39,40^. These separated measurements of mechanical phase shift, displacement and squeezing have different optimal setups, so if we work with the combination of these three processes it is difficult to obtain the optimal measurement scheme (and even to define optimality). Applying the results obtained concerning the estimation of a general Gaussian state^32,41–44^ to the output of the general Gaussian channel only yields a theoretical bound for estimation efficiency. In practice, there can be various restrictions which make this bound unattainable^45^. Within the field of optomechanics, only the estimation of coupling strength has a mathematically thorough analysis^46^.

The application of optomechanical systems to metrology and estimation theory is a rapidly developing field, as evidenced by the number of publications. In the publication by Zheng and coworkers^47^, the authors propose a sensing strategy for a quadratically coupled cavity optomechanical system. Zhao et al. considered an intracavity optical parametric amplifier as a means to achieve high precision of force sensing^48^, which extended the previous proposal by Peano et al.^49^ Schneiter et al. investigated the possibilities of optimal sensing in an optomechanical system with quadratic optomechanical coupling and a mechanical squeezer used as a resource^50^. In contrast to these works, we propose a sensing method based on the standard simple model of linearized optomechanical interaction. Our method uses short pulses to measure a mechanical force fast and does not require nonlinear interactions or mechanical squeezing, and is supposed to operate in the regime that has been proven multiple times to match with a great precision the theoretical models.

In this work we present a high-precision multiparameter estimation scheme of an unknown Gaussian process for weak force sensing. We use basic quantum nondemolition (QND) interactions in the fast stroboscopic regime, and also include realistic noises and losses peculiar to optomechanical experiments. The proposed method can be applied successfully to a large variety of basic optomechanical systems without sideband resolution^15^. We used only a coherent light mode in the cavity, which was shown to be sufficient in the case of limited interaction strength in light-matter interferometry^51^. However, in the fast stroboscopic regime of quantum optomechanics^52,53^, the light-matter interaction cannot reach even a weak beam-splitter coupling. In addition, the beam-splitter type of coupling approximation requires the resolved sideband condition to be met: the mechanical eigenfrequency must exceed the optical linewidth ($\omega _m \gg \kappa$) and also demands a precise engineering of the cavity, and therefore might be difficult to achieve experimentally. In contrast, a stroboscopic quantum nondemolition coupling is broadly available in systems with rather bad (broadband) cavities. It operates at timescales short compared to the mechanical period $2 \pi /\omega _m$, thus can detect short-time transient forces and is more resilient to the impact of the thermal environment of the mechanics^54,55^. This type of optomechanical coupling has attracted a growing theoretical interest recently regarding the fields of quantum thermodynamics and information processing^56–60^.

## Complete estimation of weak mechanical force

**Figure 1:**
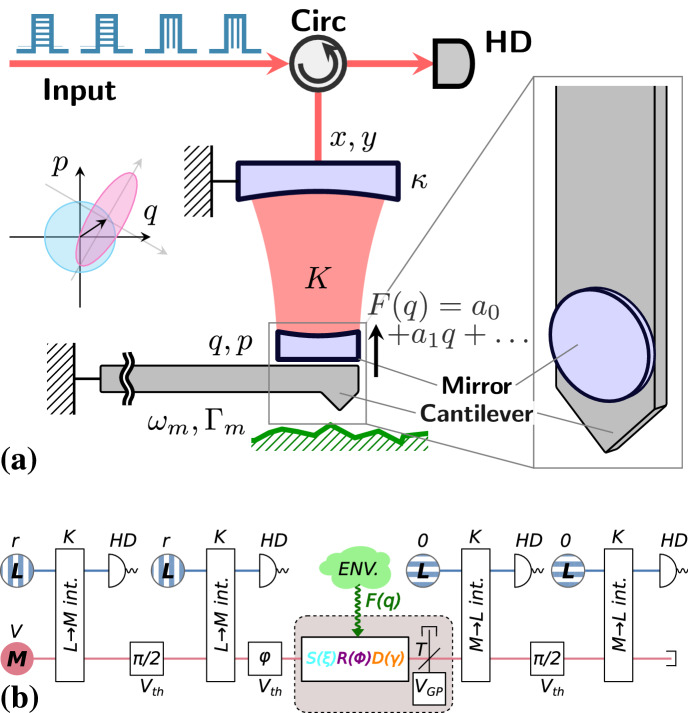
(**a**) Experimental proposal of our pulsed optomechanical scheme for the fast sensing of a small linearized mechanical force. The passive optical circulator (Circ) directs the pulses of light to the optomechanical cavity to interact with the mechanical oscillator probing the force (here: a mirror mounted on a cantilever), and then to an optical low-noise homodyne detector (HD). The deterministic part of a linearized small mechanical force described by the Gaussian unitary transformation in Eq. (1) can be provided by the interaction of the cantilever with a surface (shown in green). The inset in the frame shows an example of the physical implementation of a mirror mounted on the tip of a cantilever (inspired by Ref.^61^). (**b**) Schematic representation. A mechanical oscillator (mode *M*) in thermal state with variance *V* interacts four times with a coherent light mode (mode *L*) with amplitude *r* (for encoding, vertical stripes) or vacuum (for read-out, horizontal stripes), respectively; their interaction is approximated by a QND interaction (9) of strength *K*. Between the encoding and the readout, the matter mode can be modified by a general Gaussian process (*GP*) including loss (*T*) and noise ($V_{GP}$). The squares mark free evolution of the mechanical mode either for making the QND couplings in orthogonal directions (marked with $\pi /2$) or in preparation for having different input states of the Gaussian process (marked with $\varphi$). During these free evolutions there is also some thermalization present ($V_{th}$). The quadratures of the light are measured using HD to estimate the parameters of the Gaussian process. The mode *M* itself is inaccessible other than by an optomechanical interaction.

The proposed methodology is summarized in Fig. 1. Any weak deterministic force approximated by $F(q)\approx a_0+a_1q$, where $a_0$ and $a_1$ are real parameters, exerted upon a mechanical mode of the oscillator can be described completely by a weak Gaussian unitary transformation. As the weak Gaussian transformation is fast compared to the mechanical period, it is sufficiently close to unitary. Each possible Gaussian unitary transformation of a single mechanical mode can be decomposed into three independent Gaussian transformations^32^:

1 $$\begin{aligned} \rho ^*=R(\Phi )D(\gamma )S(\xi )\rho S(\xi )^\dagger D(\gamma )^\dagger R(\Phi )^\dagger , \end{aligned}$$

where $S(\xi )=\exp (1/2 \xi a^{\dagger 2}-1/2 \xi ^{*} a^2)$ is the squeezing operator (with $\xi =w \mathrm {e}^{i \alpha }$), $R(\Phi )=\exp (i \Phi a^{\dagger }a)$ is the phase shift operator, and $D(\gamma )=\exp (\gamma a^{\dagger }-\gamma ^* a)$ is the displacement operator (with $\gamma =d \mathrm {e}^{i \beta }$). For the mechanical oscillator, *a* and $a^{\dagger }$ are the annihilation and creation operators satisfying $[a,a^{\dagger }]=1$. Instead of estimating the real parameters $a_0$ and $a_1$ of the deterministic force, we will estimate, for the sake of completeness, each parameter of the Gaussian unitary process (1). Note that as this estimation approach can result in any Gaussian, it could detect small forces even beyond conservative ones. Finally, small mechanical losses and excess noise during the process will be estimated separately.

The displacement is described by its magnitude (*d*) and its direction $(\beta )$. The squeezing is described by its magnitude $(s=\mathrm {e}^w)$ and by its direction $(\alpha )$. The connection between the parameters of the unitary evolution (1) and the force $F(q) \approx a_0 + a_1 q$ acting on the interval $t \in [t_0, t_0 + \tau ]$, can be determined relatively easily. Assuming the force acts very quickly such that the free evolution of the mechanics can be neglected, the force causes simple transformation of quadratures:

2 $$\begin{aligned} q (t_0 + \tau ) \approx q (t_0), \quad p (t_0 + \tau ) \approx p (t_0) + a_0 \tau + a_1 \tau q (t_0). \end{aligned}$$

The Gaussian map which generates these dynamics is given by (1) with the parameters

$$\begin{aligned} \gamma&= i a_0 \tau ;\\ \tan \Phi&= a_1 \tau / 2;\\ \cosh ^2 w&= 1 + a_1 \tau / 4 ;\\ \sin \alpha&= \frac{ 2 a_1 \tau \sqrt{ \frac{ \lambda - 2 }{ \lambda + 2 }}}{ \lambda ^2 - 2 \lambda }, \end{aligned}$$

where $\lambda = \sqrt{ 4 + a_1 \tau }$. Generally, if the duration of the force action is sufficient for the free evolution to cause a noticeable effect, the simple transformations above are changed. For example, the non-negligible own restoring force $F_{spring}(q) = - \omega _m q$ of the oscillator will modify the parameter $a_1 \rightarrow a_1 - \omega _m$. This disturbance has a simple solution based on the fact that the own oscillator’s evolution is deterministic and can be subtracted in the postprocessing. For such processing, it is required that a precise calibration of the oscillator’s frequency be available, which is the case in experiments due to the high *Q*-factors of the state-of-the-art oscillators^6^. For a more detailed account of this problem, see the Supplementary information ^62^.

To detect the force, including its short-interval free evolution, we estimate all parameters of the unknown Gaussian process (1). The most interesting aspect of this is the estimation of the squeezing magnitude *s*, which needs to be clearly distinguished from the displacement effect caused purely by the parameter *d* (and is important for quantum thermodynamics^11–14^). The advantage of estimating all parameters simultaneously is that it helps to avoid any misinterpretation of the results. This makes our approach an attractive alternative to the procedures proposed earlier which estimated only the squeezing in itself.

### Stroboscopic optomechanical model

The proposed estimation of the weak external force uses the displacement of a mechanical oscillator as a probe read out by light. An optomechanical cavity is typically used to enhance coupling between the mechanical mode and a field^15^. In such a system mechanical motion is coupled to light by radiation pressure and in turn modulates the resonant frequency of the cavity. Despite the large differences in physical realizations of optomechanical systems, the majority of such systems are theoretically equivalent to a single-mode Fabry–Pérot cavity with a movable mirror^63^.

To estimate a weak mechanical force sufficiently fast, we consider a pulsed scheme in which the mechanical mode interacts very briefly and only four times with a mode of light. After the first two interactions (which can be considered as the preparation of the probe) the mechanical mode is subject to an external Gaussian process (1) with unknown properties.

The mechanical mode is described by the quadratures $q=(a^{\dagger }+a),$ and $p=i(a^{\dagger }-a)$. Using the quadratures the effect of a general Gaussian process (1) can be rewritten as a general linear process on quadrature variables:

3 $$\begin{aligned} q^*=A\cdot q+B\cdot p+\Delta q, \quad p^*=C\cdot q+D\cdot p+\Delta p, \end{aligned}$$

where $q^{*}$ and $p^*$ denote the output quadratures, $\Delta q$ and $\Delta p$ determine the displacement parameters (*d* and $\beta$), and *A*, *B*, *C*, *D* determine the phase shift ($\Phi$) and the squeezing (*s* and $\alpha$) parameters.

Note that the coefficients of a general transformation (3) are related to the parameters of the unitaries from (1) as

4 $$\begin{aligned} \Delta q= & {} \mathrm { Re } \gamma , \quad \Delta p = \mathrm { Im } \gamma , \end{aligned}$$

5 $$\begin{aligned} A= & {} \cos \Phi \cosh w + \cos ( \alpha + \Phi ) \sinh w , \end{aligned}$$

6 $$\begin{aligned} B= & {} - \cosh w \sin \Phi + \sin ( \alpha + \Phi ) \sinh w , \end{aligned}$$

7 $$\begin{aligned} C= & {} \cosh w \sin \Phi + \sin ( \alpha + \Phi ) \sinh w , \end{aligned}$$

8 $$\begin{aligned} D= & {} \cosh w \cos \Phi - \cos ( \alpha + \Phi ) \sinh w . \end{aligned}$$

The quantum state of an optical pulse is described by the quadratures *x*, *y*. If the duration of the pulse is short compared to the mechanical period ($\omega _m \tau \ll 1$) and, simultaneously, sufficiently longer than the inverse cavity linewidth ($\kappa \tau \gg 1$) then a QND interaction of such a pulse with the mechanical oscillator is close to lossless and can be approximated by equations^52,53,62,64,65^:

9 $$\begin{aligned} x^\text {out} = x^\text {in}, \quad y^\text {out} = y^\text {in}+K q, \quad q'= q, \quad p'= p + K x^\text {in}, \end{aligned}$$

with the gain $K = \sqrt{{ 2 g^2 \tau }/{ \kappa }}$, where $\kappa$ is the decay rate of the cavity, *g* is the optomechanical coupling rate. Further on, we assume that we are in the bad-cavity limit, that is, $\kappa \gg \omega _m$. Such a QND interaction maps the mechanical position (*q*) onto the phase quadrature $y^\text {out}$ of the leaking field, which can be measured. In order to obtain information regarding the mechanical momentum, one has to wait a quarter of the mechanical period $T_m$ (i.e., a $\pi /2$ rotation in the phase space) until the mechanical quadratures are, in effect, swapped. During this exchange the mechanical damping has to be taken into account:

10 $$\begin{aligned} q(T_m/4)&= \sqrt{ \eta } p(0) + \sqrt{ 1 - \eta } \delta q , \end{aligned}$$

11 $$\begin{aligned} p(T_m/4)&= \sqrt{ \eta } q(0) + \sqrt{ 1 - \eta } \delta p , \end{aligned}$$

where $\eta \equiv \exp [ - \frac{ 2 \Gamma _m \pi }{ \omega _m} ] = \exp [ - \frac{ 2 \pi }{ Q_m } ]$, $\Gamma _m$ and $Q_m$ are, respectively, the viscous damping rate and the quality factor of the mechanical oscillator. The variances of the noise can be given as^62^

12 $$\begin{aligned} \mathrm {Var}\,\delta q=\mathrm {Var}\,\delta p=2n_{th}+1=:V_{th}. \end{aligned}$$

here $n_{th} \approx \frac{ k_B \Theta }{ \hslash \omega _m }$ is the mean occupation of the bath, defined by its temperature $\Theta$.

#### Numerical parameters

Figure 1a shows a possible implementation of the discussed optomechanical scheme. Inspired by the current experiments and proposals in stroboscopic optomechanics^52–55,57,60,66^, the range of parameters we used for numerical simulations are summarized in Table 1. These works summarize the relevant experiments of the field and also some theoretical papers by experimental groups, which could serve as the base of their future work. The mechanical oscillator in such setups is represented by an oscillating mechanical semi-transparent membrane in a cavity. Unless the setup is brought into a cryogenic environment, the occupation of the bath is rather high. At the same time, we assume that it is possible to cool down the mechanical motion by a few orders of magnitude, e.g., by following the strategy proposed by Vanner et al.^53^

**Table 1:** Experimental parameters for short-pulsed optomechanical experiments inspired by Refs.^52–55,57,60,66^.

Mechanical quantity	Currently available	Under development
Bath variance $(V_{th})$	$5\cdot 10^4$	$10^3-10^4$
Initial variance(*V*)	$V_{th}/100$	$V_{th}/1000$
QND gain (*K*)	$2\cdot 10^{-4}$	$0.1-1$
Mechanical loss $(1-\eta )$	$10^{-4}-10^{-2}$	0

An important remark is that the parameters we use in our numerical simulations are selected in-between the numbers reported in columns “Currently available” and “Under development” of Table 1, which will prove the feasibility of the given method in real-life applications.

### The proposed estimation method

Our earlier results^51^ suggest that one can improve estimation efficiency by using a classical coherent light mode inside the cavity instead of a vacuum mode (note that the amplitude of this light mode *r* is not the same as that of the laser). To obtain a similar effect within the described, more realistic model, we will use the scheme from Fig. 1b, involving four QND interactions, each of which is described by Eq. (9). This QND transformation is quite simple: the *x* quadrature of the light mode is transferred to the *p* quadrature of the matter mode (which is important in the first two interactions, that is, to encode the input state of the Gaussian process), while the *q* quadrature of the matter mode is transferred to the *y* quadrature of the light mode (which is important in the last two interactions, that is, to extract the output state of the Gaussian process).

There is a time delay between subsequent QND interactions letting the state evolve in the needed position. Between the first two and the last two QND interactions there is always a delay resulting in a $\pi /2$ phase shift of the matter mode, meaning that the encoding and the extraction are made in orthogonal directions. Note that we could, in principle, use a single QND interaction with a $\pi /4$ phase shift of the matter mode (as that would also generate a displacement in both quadratures). We chose the symmetric setup presented in Fig. 1b as it can be described by simpler equations and also because it produces a larger ($\sqrt{2}$ times) displacement. The phase shift between the second QND and the unknown Gaussian process is an arbitrary $\varphi$. Let us also note that we could use an interferometric scheme (i.e., feeding the output of the pre-force light-matter interactions into the post-force interactions), but this would not significantly improve the efficiency, so we kept the simpler prepare-and-measure scheme.

**Figure 2:**
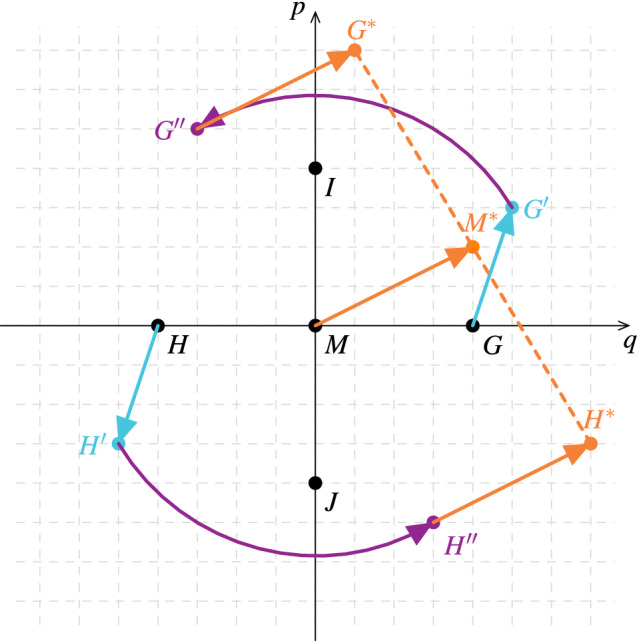
The general Gaussian transformations for input states in opposite directions (*G* and *H*). The cyan (light) lines show the effect of the squeezing, the purple (dark) lines show the effect of the phase shift, the orange (medium) lines show the effect of the displacement. Note that it is enough to plot the mean of the states, as a Gaussian transformation is a linear transformation in the phase space representation (so the mean of their transformation will be the transformation of their mean).

To understand how the estimation works, let us use in the first case an input state *G*, which lies in the direction *q* (Fig. 2). We apply a squeezing (light blue, $G'$), phase shift (purple, $G''$) and a displacement (orange, $G^*$) to it in order to get its Gaussian transformation in the phase space representation. If we perform the same transformation on the opposite input state ($H=-G$), the mean of the two output states, $M^*=\frac{G^*+H^*}{2}$, provides exactly the displacement of the Gaussian process.

On the other hand, if we only use two opposite input states (*G* and *H*), then we cannot determine the whole transformation uniquely, which is apparent from dimensional analysis. The Gaussian process has 5 real parameters ($s, \alpha , d, \beta , \Phi$), while a single input will result in an output with two real parameters. So at least 3 different input states are needed for a full, unique characterization of the unknown Gaussian process. This could be achieved also by adding two new input states (*I* and *J*) that are orthogonal to *G* and *H*. Note that mathematically, the new input states do not have to be orthogonal. Moreover, the fourth input state is not necessary, either. Nevertheless, we prefer this setup for its symmetry which also simplifies calculations.

So if we denote the outputs of the four detectors by $\mathrm {OUT}_1(\varphi )$, $\mathrm {OUT}_2(\varphi )$, $\mathrm {OUT}_3(\varphi )$, $\mathrm {OUT}_4(\varphi )$ (where $\varphi$ is the already defined quantity, which determines the additional rotation of input state before the Gaussian process), then the estimators of the displacements in directions *x* and *p* can be calculated as

13 $$\begin{aligned} {\hat{d}}_x:=\frac{\mathrm {OUT}_3(0)+\mathrm {OUT}_3(\pi )}{2 K}, \end{aligned}$$

and

14 $$\begin{aligned} {\hat{d}}_p:=\frac{\mathrm {OUT}_4(0)+\mathrm {OUT}_4(\pi )}{2 K}. \end{aligned}$$

If we already know the displacement parameter, we can subtract its value to identify only the effect of the squeezing and the phase shift:

15 $$\begin{aligned} \hat{A''_x}:=\mathrm {OUT}_3(0)/K-{\hat{d}}_x, \quad \hat{A''_p}=\mathrm {OUT}_4(0)/K-{\hat{d}}_p, \end{aligned}$$

16 $$\begin{aligned} \hat{C''_x}:=\mathrm {OUT}_3(\pi /2)/K-{\hat{d}}_x, \quad \hat{C''_p}:=\mathrm {OUT}_4(\pi /2)/K-{\hat{d}}_p. \end{aligned}$$

Using which we can estimate the other parameters of the Gaussian process:

17 $$\begin{aligned} {\hat{\Phi }}:=\arctan {\bigg (\frac{-\hat{A''_x}+\hat{A''_p}+\hat{C''_x}+\hat{C''_p}}{\hat{A''_x}+\hat{A''_p}+\hat{C''_x}-\hat{C''_p}}\bigg )}, \end{aligned}$$

18 $$\begin{aligned} {\hat{\alpha }}:=\frac{1}{2}\arctan {\bigg (\frac{\hat{A''_x}^2+\hat{A''_p}^2-\hat{C''_x} -\hat{C''_p}^2}{2(\hat{A''_x}\hat{C''_x}+\hat{A''_p}\hat{C''_p})}\bigg )}, \end{aligned}$$

19 $$\begin{aligned} {\hat{s}}:=\frac{Z+\sqrt{Z^2-4}}{2}, \end{aligned}$$

where

20 $$\begin{aligned} Z=\frac{1}{K r \sqrt{2}}\sqrt{(\hat{A''_p}+\hat{C''_x})^2+(\hat{C''_p}-\hat{A''_x})^2}. \end{aligned}$$

Let us also note that in order to maximize the efficiency of the phase shift and squeezing estimators the magnitude of the input state should be as high as possible. This means that both the strength of the light mode *r*, as well as the efficiency of its transfer to the mechanical mode should be as high as possible. Thus, the coherent light mode in the instant of first two QND interactions should be in the phase of quadrature *x*. Obviously, if there is no displacement added to the matter mode (e.g., because the light mode is in vacuum mode), the mean of the matter mode will be zero both before and after the squeezing and phase shift, so in this case only the estimation of the displacement is feasible (we will have a single input state with only two parameters).

## Numerical analysis of estimation

In the previous section we proposed an estimation scheme, while in the following we investigate in detail the efficiency of this method. To quantify estimation efficiency we use the mean squared error (MSE)

21 $$\begin{aligned} \mathrm {MSE}\,({\hat{\theta }}):=\langle ({\hat{\theta }}-\theta )^2 \rangle , \end{aligned}$$

with a fixed number of measurements (*N*), where $\theta$ is the quantity of our interest (in the figures we have $\theta \in \{s,\Phi , d\}$). The exact expected value is difficult to calculate due to the nonlinearity of the estimators, so the MSE values shown in the figures are simulation results from $10^4$ trials. So, in essence, our method assumes that our model is valid, and we numerically simulate the whole process. That is, we generate the input state and simulate the optomechanical interaction, the force applied to the mechanical mode, and the imperfections. Then from the outcome, we estimate the parameter ${\hat{\theta }}$ assuming that we do not know the parameters of the force and imperfections but do know the system’s parameters (*N*, *K*, *r*, *V*). With this method, we can emulate a real process (with an arbitrary set of parameters) and calculate the squared distance of the estimated parameter from the true value.

**Figure 3:**
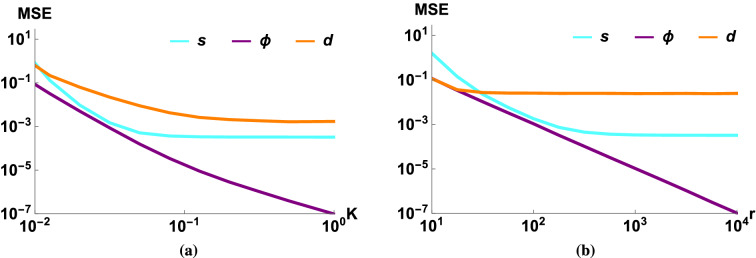
The MSE of the estimators of the Gaussian process as a function of (**a**) the transfer coefficient (*K*), and (**b**) the displacement of the light mode (*r*). The purple (dark) lines correspond to the phase shift, the orange (medium) lines correspond to the magnitude of the displacement and the cyan (light) lines correspond to the magnitude of the squeezing. The parameters were $N=10^5$, $V=10$, $T=0.99$, $1-\eta =10^{-3}$, $V_{th}=V_{GP}=10^4$, $\Phi =0.03$, $d=0.05$, $\beta =0.4$, $s=1.02$, $\alpha =-0.6$, (**a**) $r=10^2$, (**b**) $K=0.03$.

The parameters used by these numerical simulations are based on the values shown in Table 1. The transfer coefficient *K* and the amplitude of the light mode *r* are the two quantities which mainly determine the efficiency of the estimators of the parameters of the linearized force ($s, \Phi , d$). The dependence of estimation efficiency on these two parameters is quite similar (Fig. 3).

For low values of *K* or *r* the estimators give basically a random result, therefore the shape of the MSE curve is more or less incidental. If we increase the value of *K* or *r* over a critical value ($K_{crit}$ and $r_{crit}$, respectively) the MSE of the squeezing and the phase shift will start to decrease and by using Gaussian approximations we can obtain the asymptotic magnitude of

22 $$\begin{aligned} \mathrm {MSE}\,_{\Phi ,s} \sim \frac{1}{N r^2 K^4}, \end{aligned}$$

which means that the mean squared error is inversely proportional to the number of measurements (*N*), the mean photon number of the light mode ($r^2$), and to the square of the gain of added energy ($K^2$, the square comes from the fact that there are two L-M interactions during every path). The MSE is connected to the second moment, which implies a standard quantum scaling (the sensitivity is proportional to $1/\sqrt{N}$). The $r^{-2}K^{-4}$ factor in the scaling can also be accounted for intuitively as the sensitivity being inversely proportional to the magnitude of the displacement: the initial light mode is displaced by *r*, then the mechanical mode is displaced by *Kr*, and finally the measured light mode is displaced by $K^2 r$. Thus, even a moderate improvement in small values of the QND gain *K* results in a significant improvement in estimation efficiency. A similar, but not as significant effect can be seen when increasing the value of $r$ (which is typically already large to begin with); and also a smaller improvement can be achieved by simply increasing the number of measurements (*N*).

We showed (Fig. 3a) that even for a very weak Gaussian process and using realistic parameters we have a reasonable efficiency for $K \approx 10^{-2}$, and the precision increases rapidly if we use larger values of *K*. We should note that an optimal estimation of the squeezing parameter was already obtained by Schneiter et al.^50^, i.e., strictly speaking our estimation of squeezing is clearly suboptimal. The advantage of our method lies in its simplicity and wider applicability. In comparison, the typical value used in their method^50^ is $K=10^2$, so we obtained an improvement of up to 4 magnitudes weaker coupling strength, opening the possibility for a wider selection of physical applications. Also, as we will show below, our method is robust against experimental imperfections (and the ideal Heisenberg scaling rarely holds up in the presence of losses and noises), so in practice our method is optimal up to a constant factor.

We can see a similar effect (Fig. 3b) if we investigate the error as a function of the amplitude of the coherent light mode (*r*), the method works already around the value of $r\approx 10$, beyond which the estimators behave similarly as in the previous case. The exception is the estimator of the displacement *d*, which is independent of *r*, that is, increasing the strength of the light mode does not improve its estimation. Finally, let us note that for large values of *K* and *r* the error of the estimator of the magnitudes of displacement and squeezing saturate due to the unknown losses in the scheme (there is no such effect for the phases and phase-shift as the losses and noises are assumed to be phase-insensitive).

Using the proposed method the estimation of the phase shift is the most precise, but that parameter has already been investigated in various other ways, so the most important result is the estimation of a squeezing process. We obtained a relatively low critical level of parameters, therefore the method can be used in practical applications and a huge improvement in estimation efficiency can be achieved even with a moderate displacement *r* and transfer coefficient *K* (in both cases the saturation is achieved relatively quickly).

### Robustness of process estimation

In a real implementation of our optomechanical system, the mechanical mode will always interact, at least weakly, with its thermal environment. Such an interaction essentially performs an admixture of thermal noise to the quadratures of the mechanical mode. We take into account the admixture of the noise with variance $V_{th}$ and the loss $\eta$ that occurs between the optomechanical interactions, as described by Eqs. (10, 11). Furthermore, we assume that the thermal decoherence during the action of the Gaussian process also causes loss (*T*) and adds noise with variance $V_{GP}$. Note that there may be further errors (like the imperfect efficiency of the detectors or optical losses), but these could be handled within the proposed model (e.g., by eliminating their effect by a pre-calibration round or by incorporating their effect in the discussed losses or noises).

**Figure 4:**
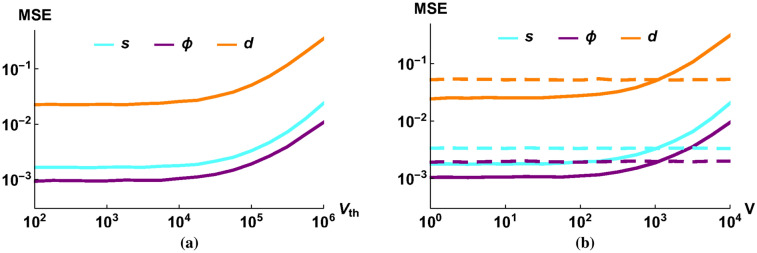
The MSE of the estimators of the Gaussian process as a function of (**a**) the thermal bath variance ($V_{th}$), (**b**) the initial variance of the mechanical mode (*V*). The purple (dark) lines correspond to the phase shift, the orange (medium) lines correspond to the magnitude of the displacement and the cyan (light) lines correspond to the magnitude of the squeezing. We have plotted for (**b**) original algorithm (solid lines) and corrected algorithm (dotted lines). The parameters are $N=10^5$, $r=10^{2}$, $K=0.03$, $T=0.99$, $1-\eta =10^{-3}$, $\Phi =0.03$, $d=0.05$, $\beta =0.4$, $s=1.02$, $\alpha =-0.6$, (**a**) $V=10$, (**b**) $V_{th}=V_{GP}=10^4$.

Since the estimators are based only on the means of the quadratures, they are not biased even if there are some added noises, because thermal noises have zero mean. Thus, the exact knowledge of $V_{th}$ and $V_{GP}$ is not important, although their value can have an effect on estimation efficiency (Fig. 4a): if the bath temperature is above a threshold, the variance of our estimators starts to gradually increase. A very similar effect (Fig. 4b, solid lines) can be observed for the initial variance of the mechanical mode (*V*), which acts also as a noise.

In the following, we explain in detail the effects of each imperfection and our proposed approach to handling them.

#### The fluctuation of the transfer coefficient* K*

The previous calculations are based on the assumption that the transfer coefficient is a constant *K* for all four QND processes for the whole time of the experiment. To investigate the effect caused by fluctuations of the transfer coefficients we introduce a simple Gaussian model

23 $$\begin{aligned} K_i(t) = K + d K_i(t), \text { with } d K_i(t)\sim {\mathscr {N}}(\Delta K_i,\delta K_i), \end{aligned}$$

where $K_i(t)$ denotes the transfer coefficient of *i*-th QND interaction ($i \in \{1,2,3,4\}$) at time *t*. This approximation assumes that the transfer coefficients’ values have a time-independent, independent (meaning that they are independent in both *t* and *i*) and identically distributed normal distribution (i.e., $K_i(t)$ has the same bias and variance for all values of *t*), with a given bias ($\Delta K_i$) and variance ($\delta K_i$) for each process QND interaction.

**Figure 5:**
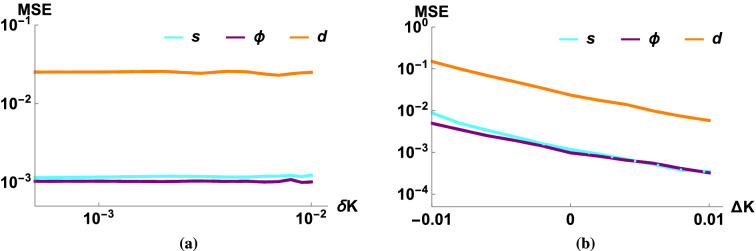
The MSE of the estimators of the Gaussian process as a function of (**a**) the variance of the transfer coefficient ($\delta K$), (**b**) the bias of the transfer coefficient ($\Delta K$). The purple (dark) lines correspond to the phase shift, the orange (medium) lines correspond to the magnitude of the displacement and the cyan (light) lines correspond to the magnitude of the squeezing. The parameters are $N=10^5$, $r=10^{2}$, $K=0.03$, $T=0.99$, $1-\eta =10^{-3}$, $\Phi =0.03$, $d=0.05$, $\beta =0.4$, $s=1.02$, $\alpha =-0.6$, $V=10$, $V_{th}=V_{GP}=10^4$, (**a**) $\forall i$: $\Delta K_i$=0, $\delta K_i=\delta K$ (**b**) $\forall i$: $\delta K_i=0.003$, $\Delta K_2=\Delta K_3=\Delta K_4=0$, $\Delta K_1=\Delta K$.

Figure 5a shows that our method is not sensitive to the variance of the transfer coefficient. Also, comparing the values to the case of constant *K* (Fig. 4), we can observe similar efficiencies, so our method performs similarly for a heavily fluctuating process and the only important quantity seems to be the mean of the transfer coefficient. This behavior would be expected if our parameter estimators were linear (due to the linearity of the expected value), but they are not. These results suggest, however, that they could be efficiently linearized regarding the transfer coefficient *K*.

On the other hand, if we change the mean of one of transfer coefficient for one QND interaction (without changing the given parameter in our estimators), that results in a biased estimation of parameters, and a change of MSE. The change depends on the exact parameter values, but in the investigated regime of parameters the estimators are not very sensitive to the bias (Fig. 5b). So we can conclude that we can have reasonably good parameter estimates even for moderately precise estimates of the mean of transfer coefficient *K*. As there exist methods to obtain this coefficient for a given experiment, the idealistic estimation method introduced above seems to be robust enough to be applicable in practice (one does not need to worry about the fluctuation of transfer coefficients).

#### Correction for very noisy matter mode

The light-matter interaction does not only contribute a displacement to the mechanical mode, but the state of the mechanical mode is partially transferred to the light mode, too. If the initial thermal state of the matter mode corresponds to a high temperature (meaning its variance *V* is large), then the given method will provide very imprecise estimates (Fig. 4b, solid lines). We can obtain better results if we notice that by the QND interaction, not only the displacement is transferred partially to the matter mode, but the initial state of matter mode is also partially transferred to the light mode. By using the two orthogonal measurements of these sequential light modes one can obtain an unbiased estimate of the initial matter mode. Then if we incorporate this bias in the Gaussian process, we can in principle improve our estimation.

Importantly, so far all the transformations were described only by their means, so as simple point-to-point transformations. In practice, however, the input state *A* is not exactly there as in the picture, it is only the mean of the input state and has a Gaussian variance around it. If this variance is large, the initial states could be really far from this mean, meaning that if we have only a finite number of data points, the results are somewhat incidental, resulting in the described increased variance. What we can do is to estimate the input state pointwise and subtract this estimate from the initial state also pointwise. This means that if the initial state ${\overline{A}}=A+D$ was far from the mean *A*, we can estimate the thermal state *D* by ${\tilde{D}}$, and ${\overline{A}}-\tilde{D}$ will be already close to *A*. The problem is that the estimation of *D* (${\tilde{D}}$) is not exact due to fundamental quantum properties. So if *D* is actually close to zero, then using this correction instead of having a more precise estimation of *A*, we will just add some noise with the correction by $\tilde{D}$. So it is only useful for large variances as it can be seen in Fig. 4b.

Let us also note that in practice we do not correct the input states, we have the outputs of detectors instead. That is, instead of the input states, we have to apply the above discussed correction to the output states. If the effect of the unknown Gaussian process is large then it results in a cumbersome problem since different input states can be translated to completely different output states. Then instead of having our simple three input state-three output state model with a simple inversion formula, we have a different formula for every input-output pair. So the essence of our simple estimation formulas will not be applicable anymore, more complicated maximum-likelihood estimators are needed in that case. However, if the effect of the Gaussian process is close to identity (which is probably close to reality if we are using short pulses and very limited interaction time), then we can simply assume that the Gaussian process is the identity operator, thus, the correction of the output states can be translated in a straightforward manner to the input states. This will introduce some bias to the estimation, but this bias will be small if the Gaussian process is close to identity.

Mathematically this means that instead of simply using $\mathrm {OUT}_3(\varphi )$ and $\mathrm {OUT}_4(\varphi )$ we use

24 $$\begin{aligned} \mathrm {OUT}_3(\varphi )-\cos \varphi \cdot (\mathrm {OUT}_2(\varphi )-K^2 r)+\sin \varphi \cdot \mathrm {OUT}_1(\varphi ) \end{aligned}$$

and

25 $$\begin{aligned} \mathrm {OUT}_4(\varphi )+\sin \varphi \cdot (\mathrm {OUT}_2(\varphi )-K^2 r)+\cos \varphi \cdot \mathrm {OUT}_1(\varphi ), \end{aligned}$$

respectively.

And if we include the measurement results of these light modes (before applying the Gaussian process) also into our estimation, we can eliminate the negative effect of high variances (Fig. 4b, dashed lines).

#### Estimation of the loss of the Gaussian process

On the other hand, the estimation of the losses is crucial and we can estimate *T* (which can be even far from unity) with the already discussed setup.

Previously we always assumed that the Gaussian process is an ideal unitary single-mode process, that is, it can be constructed by the superposition of a squeezing, a displacement and a phase shift. Here we assume the simplest imperfection, namely that during the Gaussian process there is a loss and also an additional noise. Since we are using the means for our estimations, the effect of the noise is insignificant: it will not add any bias, only the variance of the estimator will increase.

The loss, however, can cause a significant bias in our estimation. Luckily, we can estimate it from our already obtained three input states. Using the earlier notations, we can estimate of the loss during the Gaussian process as

26 $$\begin{aligned} {{\hat{T}}}=(Z^2-W)/2, \end{aligned}$$

where *Z* is defined in Eq. (13) and

27 $$\begin{aligned} W=\frac{1}{2 K^2 r^2}\bigg (\hat{A''_p}^2+\hat{C''_x}^2+\hat{C''_p}^2+\hat{A''_x}^2\bigg ). \end{aligned}$$

The derivation of this formula is rather technical: if we include into our model the loss parameter *T*, then there are altogether 6 output parameters, while the Gaussian process contains only 5 parameters plus the loss *T* (which act as the 6 variables in a system of 6 non-linear equations). From that we can express *T* as a function of the measurement outputs and after some rearrangement we can derive the simple formula (26).

Using this loss, we can correct Eqs. (13)–(19) easily:

28 $$\begin{aligned} {\hat{d}}_x:= & {} \frac{\mathrm {OUT}_3(0)+\mathrm {OUT}_3(\pi )}{2 K \sqrt{{{\hat{T}}}}}, \end{aligned}$$

29 $$\begin{aligned} {\hat{d}}_p:= & {} \frac{\mathrm {OUT}_4(0)+\mathrm {OUT}_4(\pi )}{2 K \sqrt{{{\hat{T}}}}}, \end{aligned}$$

30 $$\begin{aligned} {\hat{s}}:= & {} \frac{1}{2} \bigg (\frac{Z}{\sqrt{{{\hat{T}}}}}+\sqrt{\frac{Z^2}{{{\hat{T}}}}-4}\bigg ), \end{aligned}$$

while the two Eqs. (17)–(18) for obtaining angles remain unchanged. Note that the estimators of the displacement and squeezing are almost identical to the noiseless case (13)–(19), with a difference of a correction factor of the loss $\sqrt{{{\hat{T}}}}$. The more interesting fact is that we can estimate the loss without changing our system (this is nontrivial due to the nonlinearity of the equations), only an extra Eq. (26) is added, so we can perform an error correction using simple numerical postprocessing.

We can conclude that the efficiency of the estimators does not strongly depend on the value of *T*, that is, if we know the magnitude of the loss, then its effect can be eliminated almost perfectly. The loss during the thermalization will be minimal due to the very limited evolution of the mechanical system (less than one period), meaning that there is no need to estimate it and practically there will be no loss of the signal ($\eta \approx 1$). However, this is only an approximation which leads ultimately to mildly biased estimators, which cause the saturations in Fig. 3 (the level of saturation mainly depends on the parameter $1-\eta$).

## Discussion

To advance mechanical force sensing, we proposed a fast stroboscopic optomechanical scheme with weak coupling strength, which still allows to simultaneously and precisely estimate all parameters of an arbitrary linearized force acting on the mechanical probe. The main point of the method is to use classical coherent light modes to displace mechanical ones, so that the mean of the setup output (3) will not only depend on the displacement parameters, but on each parameter of the force. So it is possible to perform a complete characterization by only using the means of the output light mode quadratures, providing a simple, yet widely applicable estimation method for different quantum sensing applications.

We obtained from the means a multiparameter estimation with a good efficiency even using current state-of-the-art technical conditions. Similar methods were already obtained earlier^50^, but our approach is novel since it provides efficient parameter estimation even for orders of magnitude weaker optomechanical coupling than in earlier works. We showed that our proposed estimation of the mechanical squeezing process can be enhanced significantly by using coherent light in the cavity (Fig. 3). Moreover, the method tolerates even a high initial variance of the mechanical mode (Fig. 4). Finally, we showed that the enhanced estimators are robust against limited efficiency of the optical read out and other expected experimental imperfections.

Thus, by using only coherent states of light, very weak light-matter interactions and measured mean values by homodyne detectors, we have obtained complete, very robust and precise estimators of an unknown linearized mechanical force, which can be applied immediately to short-pulsed optomechanical systems^52–55,57,60^ as the current technology is already capable of providing the first proof-of-principle test of our method. They can be directly used to reliably estimate the weak squeezing in mechanical processes, which are important for the development of, e.g., quantum thermodynamics with mechanical systems^11–14^. As quantum squeezing of light can bring such mechanical sensing beyond the domain of classical optical states, our method simultaneously provides a benchmark for future investigation. Furthermore, our method could be generalized to estimate nonlinear mechanical forces using the expansion of the nonlinear function *F*(*q*).

## Supplementary Information

Supplementary Information.
