# Supplementary material for: High-precision multiparameter estimation of mechanical force by quantum optomechanics
Source: Sci Rep. 2022 Sep 26;12:16022. doi: 10.1038/s41598-022-20150-6 (PMC9512796; doi:10.1038/s41598-022-20150-6)
Supplement: Supplementary file 1 — Supplementary Information. [file 41598_2022_20150_MOESM1_ESM.pdf]

Supplemental material: High-precision multiparameter estimation of mechanical force by quantum optomechanics

Input-output relations for an optomechanical system

In this supplementary material we follow the treatment of Refs.[1–3] and derive the input-output relations for the light-matter interaction in the optomechanical system.

An optomechanical system can be considered as a Fabry–Pérot cavity with a movable mirror (cf. Fig. 1(a) of the main text). The mechanical excursions from the equilibrium point modulate the resonant frequency ω_{cav} of the cavity giving rise to the radiation-pressure-induced coupling[4], so that the overall Hamiltonian reads in the frame rotating at the cavity frequency ($\hbar = 1$):

$$H_0 = -g_0 q c^\dagger c + \tfrac{1}{4}\omega_m(p^2 + q^2), \tag{1}$$

where c ($c^\dagger$) is the annihilation (creation) operator of the intracavity light mode, q (p) position (momentum) of the mechanical oscillator with eigenfrequency ω_m. The optomechanical coupling is characterized by the so-called single-photon coupling rate g_0. Typically, this coupling is weak and has to be enhanced by a strong classical optical pump in order to produce visible results. In this paper we consider a strong resonant pump at the cavity frequency ω_{cav}.

We start with Heisenberg–Langevin equations that take into account the cavity dissipation. The characteristic duration of the optomechanical interaction in our scheme is much shorter than the mechanical period which allows to ignore the free oscillator Hamiltonian (last term in (1)). Given the excellent quality ($Q \gtrsim 10^8$) of the state-of-the-art mechanical devices[5], the mechanical decoherence is negligible over the duration of a few mechanical oscillators. Therefore, we can also ignore the mechanical dissipation for the duration of the optomechanical interaction.

$$\dot{c} = i g_0 c q - \kappa c + \sqrt{2\kappa}\,\tilde{c}^{\text{in}}, \tag{2}$$

$$\dot{p} = g_0 c^\dagger c, \quad \dot{q} = 0, \tag{3}$$

where $\tilde{c}^{\text{in}}$ is the optical input to the cavity. In our case of interest, this input consists of a strong classical drive $C^{\text{in}}(t)$ and quantum noise $c^{\text{in}}(t)$:

$$\tilde{c}^{\text{in}}(t) = C^{\text{in}}(t) + c^{\text{in}}(t). \tag{4}$$

The strong drive, represented by complex-valued function $C^{\text{in}}(t)$, can be fully controlled in the experiment and creates a classical displacement in the intracavity field. Consequently, the intracavity field can be written as a sum of a classical part C and the quantum noise δc. Substituting expressions for c and $\tilde{c}^{\text{in}}$ into (2) yields

$$\frac{d}{dt}\Big[C + \delta c\Big] = i g_0 (C + \delta c) q - \kappa (C + \delta c) + \sqrt{2\kappa}(C^{\text{in}} + c^{\text{in}}), \tag{5}$$

We can separate this equation into two, for the classical and quantum parts (where we keep the notation c for δc for brevity):

$$\dot{C} = -\kappa C + \sqrt{2\kappa} C^{\text{in}}, \tag{6}$$

$$\dot{c} = i g_0 C q - \kappa c + \sqrt{2\kappa} c^{\text{in}} + i g_0 c q. \tag{7}$$

Eq. (6) admits a solution in the form

$$C(t) = C(0)e^{-\kappa t} + e^{-\kappa t}\sqrt{2\kappa}\int_0^t \mathrm{d}s\, C^{\text{in}}(s) e^{\kappa s}. \tag{8}$$

The equation for p reads:

$$\dot{p} = g_0 |C|^2 + g_0 C (c^\dagger + c) + g_0 c^\dagger c. \tag{9}$$

For the next step, we notice that the term $g_0|C|^2$ contributes a fully deterministic classical shift of the oscillator's momentum that can be compensated by a feedback or taken an account of in processing the measurement results. This term can thus be omitted. In addition, assuming weak single-photon coupling rate g_0 (perfectly justified for most present experiments) and

strong drive $|C| \gg 1$ allows to ignore the nonlinear quadratic terms in the equations of motion (7,9). As a result, we have equations for c and p:

$$\dot{c} = ig(t)q - \kappa c + \sqrt{2\kappa}c^{\text{in}}, \tag{10}$$
$$\dot{p} = g(t)X, \tag{11}$$

where we denote

$$X = c + c^{\dagger}, \quad g(t) = g_0 C(t). \tag{12}$$

The solution to (10) is

$$c(t) = e^{-\kappa t}\left[c(0) + iq(0)\int_0^t \mathrm{d}s\, g(s)e^{\kappa s} + \sqrt{2\kappa}\int_0^t \mathrm{d}s\, c^{\text{in}}(s)e^{\kappa s}\right]. \tag{13}$$

Recast to the quadratures in a standard manner, $X = c + c^{\dagger}$ and $P = i(c^{\dagger} - c)$, this is equivalent to

$$X(t) = X(0)e^{-\kappa t} + \sqrt{2\kappa}\int_0^t \mathrm{d}s\, X^{\text{in}}(s)e^{-\kappa(t-s)}, \tag{14}$$
$$P(t) = P(0)e^{-\kappa t} + \sqrt{2\kappa}\int_0^t \mathrm{d}s\, P^{\text{in}}(s)e^{-\kappa(t-s)} + q(0)\int_0^t \mathrm{d}s\, g(s)2e^{-\kappa(t-s)}. \tag{15}$$

Finally, the leaking light can be derived from the input-output relation for the field

$$c^{\text{out}} = -c^{\text{in}} + \sqrt{2\kappa}c. \tag{16}$$

To derive the quadratures of the detected light this expression has to be substituted into

$$c^{\text{det}} = \int_0^t \mathrm{d}s\, f^{\text{det}}(s)c^{\text{out}}(s), \tag{17}$$

where f^{det} is the detection filter function, defined by the temporal local oscillator field profile. For the commutation relations to preserve, this function has to satisfy

$$\int_0^t |f^{\text{det}}(s)|^2 \mathrm{d}s = 1. \tag{18}$$

Combining the definitions, after some algebra, we obtain for the quadratures of the leaking light

$$P^{\text{out}} = \int_0^t \mathrm{d}s\, P^{\text{in}}(s)\left[-f^{\text{det}}(s) + 2\kappa\int_s^t \mathrm{d}\xi\, f^{\text{det}}(\xi)e^{-\kappa(\xi-s)}\right] + \sqrt{2\kappa}P(0)\int_0^t \mathrm{d}s\, f^{\text{det}}(s)e^{-\kappa s}$$
$$+ 2\sqrt{2\kappa}q(0)\int_0^t \mathrm{d}\xi\, g(\xi)\int_\xi^t \mathrm{d}s\, f^{\text{det}}(s)e^{-\kappa(s-\xi)}, \tag{19}$$
$$X^{\text{out}} = \int_0^t \mathrm{d}s\, X^{\text{in}}(s)\left[-f^{\text{det}}(s) + 2\kappa\int_s^t \mathrm{d}\xi\, f^{\text{det}}(\xi)e^{-\kappa(\xi-s)}\right] + \sqrt{2\kappa}X(0)\int_0^t \mathrm{d}s\, f^{\text{det}}(s)e^{-\kappa s} \tag{20}$$

In these equations we can define the input optical mode quadratures as

$$x_{\text{o}}^{\text{in}} = \int_0^t \mathrm{d}s\, X^{\text{in}}(s)\frac{f_{\text{o}}^{\text{out}}(s)}{\left[\int_0^t \mathrm{d}\xi\, |f_{\text{o}}^{\text{out}}(s)|^2\right]^{1/2}}; \text{ where } f_{\text{o}}^{\text{out}} = \left[-f^{\text{det}}(s) + 2\kappa\int_s^t \mathrm{d}\xi\, f^{\text{det}}(\xi)e^{-\kappa(\xi-s)}\right]. \tag{21}$$

Then (20) transforms into

$$X^{\text{out}} = T_{\text{in}}x_{\text{o}}^{\text{in}} + \sqrt{2\kappa}X(0)\int_0^t \mathrm{d}s\, f^{\text{det}}(s)e^{-\kappa s}, \text{ where } T_{\text{in}} = \left[\int_0^t \mathrm{d}\xi\, |f_{\text{o}}^{\text{out}}(s)|^2\right]^{1/2}. \tag{22}$$

On the side of the mechanics, the solution to (11) is

$$p(t) = p(0) + \int_0^t \mathrm{d}s\, g(s)X(s) = p(0) + X(0)\int_0^t \mathrm{d}s\, g(s)e^{-\kappa s} + \sqrt{2\kappa}\int_0^t \mathrm{d}s\, X^{\text{in}}(s)\int_s^t \mathrm{d}\xi\, g(\xi)e^{-\kappa(\xi-s)}$$
$$= p(0) + X(0)\int_0^t \mathrm{d}s\, g(s)e^{-\kappa s} + K_p x_{\text{m}}^{\text{in}}, \tag{23}$$

where we have defined the input mode $x_{\rm m}^{\rm in}$ and the transfer gain K_p:

$$x_{\rm m}^{\rm in} = \int_0^t {\rm d}s\, X^{\rm in}(s) \frac{f^{\rm in}(s)}{\left[\int_0^t {\rm d}\xi\, |f^{\rm in}(s)|^2\right]^{1/2}}; \qquad f^{\rm in}(s) \equiv \sqrt{2\kappa}\int_s^t {\rm d}\xi\, g(\xi) e^{-\kappa(\xi - s)}, \tag{24}$$

$$K_p = \left[\int_0^t {\rm d}\xi\, |f^{\rm in}(s)|^2\right]^{1/2} \tag{25}$$

Note, that in the general case of an arbitrary driving profile, the quadrature $x_{\rm m}^{\rm in}$ is not the same as $x_{\rm o}^{\rm in}$.

Together with trivial $q(t) = q(0)$, Eqns. (19,20,23) represent the full set of input-output relations describing our optomechanical interaction. Apart from the standard linearization approximation, these equations are exact. To have them fully defined to the end, one has to specify concrete temporal shapes of the drive $C^{\rm in}(t)$ and the detection profile $f^{\rm det}(t)$. A certain choice of the driving profile $C^{\rm in}(t)$ selects the concrete temporal modes of the input light that couple most efficiently to the mechanical mode and also to the leaking light. In other words, this selection can be viewed as a tuning of the filtering of the quantum information that is transferred from the input light onto the mechanics.

Formally, after one has defined the drive profile $C^{\rm in}(t)$, the coupling strength profile $g(t)$ is defined as well, which defines the profiles of the input modes. A choice of $f^{\rm det}$ then fixes the temporal profile of the output optical mode. The choice of the detection profile can be arbitrary, however, given the task of effective reconstruction of $q(0)$, a reasonable choice is such that maximizes the multiplier of $q(0)$ in $P^{\rm out}$.

The derived input-output equations have a form similar to the QND coupling. The differences are caused mostly by the presence of the intracavity mode which serves as a mediator for the interaction between the light outside the cavity and the mechanical oscillator. This mode has a threefold effect on the input-output relations. First, its vacuum noise is admixed to the output states of both light and mechanics. Second, it brings an asymmetry into the transformations which is equivalent to adding extra noise terms, originating from the input light, to either output light or mechanical mode. Thanks to the input light being in vacuum, this is also an additional source of vacuum noise. Third, it can slightly reduce the transformation gain K. Each of this effects can be absorbed within the model of the main text with extra mechanical noise and a reduced K.

It is instructive to note at this stage, that in the relevant parameter regime, where the pulses are longer than the inverse cavity linewidth, so that $\kappa t \gg 1$,

$$e^{-\kappa t} \approx 0, \quad e^{-\kappa(t-s)} \approx \delta(t-s), \tag{26}$$

and consequently

$$C(t) \approx \sqrt{2\kappa}\int_0^t {\rm d}s\, C^{\rm in}(s)\delta(t-s) = \sqrt{\frac{2}{\kappa}} C^{\rm in}(t). \tag{27}$$

Assuming constant drive strength $C^{\rm in}(s) = C^{\rm in}, 0 \le s \le t$ yields a constant coupling strength $g(t) = g$. After carefully performing the algebraic transformations and putting $f^{\rm det}(s) = \frac{1}{\sqrt{t}}$, we arrive to the symmetric QND interaction of the main text where

$$\begin{pmatrix} x^k \\ y^k \end{pmatrix} = \frac{1}{\sqrt{t}}\int_0^t {\rm d}s \begin{pmatrix} X^k(s) \\ Y^k(s) \end{pmatrix}, \quad k = \text{in,out}. \tag{28}$$

Joint influence of the external force and free evolution of the mechanical system

In the main text we focus on the fast weak force that acts on timescales insufficient for the own evolution of the mechanical oscillator to play a significant role. To account for this, here we show how the free evolution changes the dynamics caused solely by the external force.

We consider here a noiseless case of the mechanical evolution which is justified for short-time dynamics of duration $\tau \ll \omega_m^{-1}$ and, moreover, the thermal noise during the Gaussian process is captured by an admixture of the noise $V_{\rm GP}$ in the main text. The Langevin equations for the mechanical oscillator read in presence of the signal force

$$\dot q = \omega_m p, \quad \dot p = (a_1 - \omega_m) q + a_0 \equiv b_1 q + a_0 \tag{29}$$

with the solution

$$q(\tau) = q_0 \cosh\sqrt{b_1\omega_m}\tau + p_0\sqrt{\frac{\omega_m}{b_1}}\sinh\sqrt{b_1\omega_m}\tau + \frac{2a_0}{b_1}\sinh^2\left(\frac{1}{2}\sqrt{b_1\omega_m}\tau\right) \tag{30}$$

$$p(\tau) = q_0\sqrt{\frac{b_1}{\omega_m}}\sinh(\sqrt{b_1\omega_m}\tau) + p_0\cosh\sqrt{b_1\omega_m}\tau + \frac{a_0}{\sqrt{b_1\omega_m}}\sinh\sqrt{b_1\omega_m}\tau. \tag{31}$$

These transformations, being generated by a Gaussian evolution, have a form described by Eq.(6) of the main text. Consequently, it is possible to perform an estimation of the parameter b_1 from which, having a precisely calibrated value of the frequency ω_m, it is possible to obtain the value of a_1.

Free evolution of the mechanical oscillator

To derive the Eqs. (13,14) of the main text we consider the free damped evolution of the harmonic oscillator, which can be described by the following quantum Langevin equations

$$\dot{q} = \omega_m p, \quad \dot{p} = -\omega_m q + \sqrt{2\gamma}\xi^{\text{th}} - \gamma p, \tag{32}$$

where γ is the viscous damping rate of the mechanics, ξ^{th} the thermal noise operator. The latter obeys

$$\tfrac{1}{2}\left\langle \xi^{\text{th}}(t)\xi^{\text{th}}(t') + \xi^{\text{th}}(t')\xi^{\text{th}}(t)\right\rangle = (2n_{\text{th}}+1)\delta(t-t') \text{ and } \left[\xi^{\text{th}}(t), q(t')\right] = i\sqrt{2\gamma}. \tag{33}$$

Integration of Eq. (32) yields

$$q(t) = e^{-\frac{\gamma t}{2}}\left(\left[\cos\omega_m\sigma t + \varepsilon\sin\omega_m\sigma t\right]q(0) + \frac{1}{\sigma}\sin\omega_m\sigma t\, p(0) + \Delta q(t)\right), \tag{34}$$

$$p(t) = e^{-\frac{\gamma t}{2}}\left(\left[\cos\omega_m\sigma t - \varepsilon\sin\omega_m\sigma t\right]p(0) - \frac{1}{\sigma}\sin\omega_m\sigma t\, q(0) + \Delta p(t)\right), \tag{35}$$

where $\sigma = \sqrt{1-(\gamma/2\omega_m)}$ and $\varepsilon = \gamma/(2\omega_m\sigma)$. The noise operators Δq and Δp are defined as

$$\Delta q(t) = \sqrt{2\gamma}\int_0^t \mathrm{d}t'\, e^{\gamma t'/2}\frac{1}{\sigma}\sin\omega_m\sigma(t-t')\xi^{\text{th}}(t'), \tag{36}$$

$$\Delta p(t) = \sqrt{2\gamma}\int_0^t \mathrm{d}t'\, e^{\gamma t'/2}\left[\cos\omega_m\sigma(t-t') - \varepsilon\sin\omega_m\sigma(t-t')\right]\xi^{\text{th}}(t') \tag{37}$$

Recently the fabrication of the micro- and nanomechanical oscillators allowed to achieve Q factors as high as $Q = \frac{\omega_m}{\gamma} > 10^8$ [5,6], therefore we can use approximations $\sigma \approx 1$, $\varepsilon \approx 0$. Thereby for the evolution over a quarter of the mechanical period $t = T_m/4$, with a precision up to $O(Q^{-1})$ we arrive at Eqs. (13,14) of the main text, where

$$\delta q = \int_0^\tau \mathrm{d}t\, e^{\gamma t/2}\sin\omega_m(\tau - t)\xi^{\text{th}}(t), \quad \delta p = \int_0^\tau \mathrm{d}t\, e^{\gamma t/2}\cos\omega_m(\tau - t)\xi^{\text{th}}(t). \tag{38}$$

References

1. Vanner, M. R. *et al.* Pulsed quantum optomechanics. *Proc. Natl. Acad. Sci.* **108**, 16182–16187 (2011).
2. Aspelmeyer, M., Kippenberg, T. J. & Marquardt, F. Cavity optomechanics. *Rev. Mod. Phys.* **86**, 1391–1452 (2014).
3. Vostrosablin, N., Rakhubovsky, A. A., Hoff, U. B., Andersen, U. L. & Filip, R. Quantum optomechanical transducer with ultrashort pulses. *New J. Phys.* **20**, 083042 (2018).
4. Law, C. K. Interaction between a moving mirror and radiation pressure: A Hamiltonian formulation. *Phys. Rev. A* **51**, 2537–2541 (1995).
5. Norte, R., Moura, J. & Gröblacher, S. Mechanical Resonators for Quantum Optomechanics Experiments at Room Temperature. *Phys. Rev. Lett.* **116**, 147202 (2016).
6. Yuan, M., Cohen, M. A. & Steele, G. A. Silicon nitride membrane resonators at millikelvin temperatures with quality factors exceeding 108. *Appl. Phys. Lett.* **107**, 263501 (2015).
